# Influence of High Expectations and Selfie Photographs on the Satisfaction Level of Facial Plastic Surgery in the Eastern Region of Saudi Arabia

**DOI:** 10.7759/cureus.47087

**Published:** 2023-10-15

**Authors:** Abdulaziz Abaalkhail, Abdulrahman Alhadlaq, Mohammed Albesher, Mohammed Al Molhim, Hassan Aldandan, Zahra Alali

**Affiliations:** 1 Plastic Surgery, Security Forces Hospital, Riyadh, SAU; 2 General Practice, King Faisal University, Al-Ahsa, SAU; 3 Ophthalmology, King Faisal University, Al-Ahsa, SAU; 4 Medicine, King Faisal University, Al-Ahsa, SAU

**Keywords:** facial surgery, satisfaction, expectation, selfie, plastic surgery

## Abstract

Introduction

Selfies are most commonly posted on social media, where their popularity has led to intensified awareness by the subjects of their appearance. This, led to higher demand for cosmetic procedures, intending to enhance how the subject appears in future selfies.

Aim

This study aims to evaluate the influence of high expectations and selfie photographs on satisfaction level results of facial plastic surgery in Eastern Province, Saudi Arabia.

Subject and methods

This cross-sectional study was conducted among Eastern Province residents of Saudi Arabia. A self-administered questionnaire was sent to the residents using an online survey. The questionnaire included demographic characteristics (i.e., age, gender, nationality, etc.) and specific questions regarding the influence of selfie photographs and high expectations on the satisfaction rates among patients who had facial plastic surgery.

Results

Of the 192 participants, 79.2% were females, and 44.8% were aged between 18 to 30 years old. The most commonly sought cosmetic surgery was rhinoplasty (38.5%). 71.4% were taking selfies, while 39.1% perceived better expectations with plastic surgery. High expectations with plastic surgery were more prevalent in older and married participants. It is important to note that an increased satisfaction rate after cosmetic procedures was more associated with high expectations of plastic surgery.

Conclusion

High expectations directly influence satisfaction with appearance after the cosmetic procedure, but taking selfies does not. Respondents of older age and who were married demonstrated better expectations of plastic surgery compared to the rest of the groups. More investigations are required to confirm the relationship between expectation and taking selfies in terms of satisfaction with self-appearance after cosmetic surgery.

## Introduction

Cosmetic surgery is a specialized field that focuses on improving or altering an individual's physical appearance through surgical and therapeutic techniques. In the past few years, there has been a notable increase in the global demand for cosmetic procedures [1]. Based on information from the American Society of Plastic Surgery (ASPS), there were about 17 million cosmetic procedures performed in 2016. These procedures had a total annual cost of approximately 16.4 billion USD [2]. As mentioned in a study performed by the International Society of Aesthetic Plastic Surgeons (ISAPS), Saudi Arabia holds the 29th position out of the top 30 countries worldwide with the highest rates of cosmetic procedures [3]. Both men and women have witnessed a 58% increase in cosmetic surgeries from 2012 to 2016 [4]. The majority of these procedures (92%) were performed on females, with rhinoplasty being one of the top five procedures from 2015 to 2016 [2].

Several factors contribute to the growing trend of facial plastic surgery, including media influence, medical advancements, and patient characteristics [5]. Exposure to media, particularly through satellite television, has been identified as a contributing factor to the higher rates of rhinoplasty [6]. Studies have shown that increased media exposure, low self-esteem, and dissatisfaction with life can increase the likelihood of undergoing plastic surgery [7]. Furthermore, the ease of interacting with surgeons and seeking consultations through social media has contributed to a surge in patients seeking plastic surgery [8,9]. In 2019, 72% of practitioners from the American Academy of Facial Plastic and Reconstructive Surgeons reported an increase in patients requesting cosmetic procedures to enhance their appearance in selfies, representing a 15% rise from the previous year [10]. The prevalence of taking and sharing selfies on platforms like Snapchat and Facebook has also increased significantly [11]. This heightened awareness of one's appearance in selfies has led to a greater demand for cosmetic surgery aimed at improving future selfie images.

A study by Ward et al. found that nasal size appears larger by 30% in selfies, which may contribute to the increased demand for rhinoplasty [12]. Another study revealed that patients with a history of cosmetic rhinoplasty had higher rates of depression, body dissatisfaction, and lower self-esteem compared to those without such a history [13]. However, it is important to note that high expectations and reliance on selfie photographs can sometimes result in unsatisfactory outcomes of facial enhancements. Therefore, the aim of this research is to investigate the influence of high expectations and selfie photographs on the satisfaction level of facial plastic surgery in Saudi Arabia.

## Materials and methods

This cross-sectional study was performed for six months from October 2022 to March 2023. It was conducted among the adult population (≥ 18 years) in Eastern Province, Saudi Arabia. The data was collected using a convenient sampling technique, through a self-administered questionnaire as the main data collection method.

For this study, certain inclusion and exclusion criteria have been established. Inclusion criteria consist of individuals who are at least 18 years old and residents of the Eastern Province, Saudi Arabia. The exclusion criteria encompass those who declined to participate in the study, did not fully complete the questionnaire, are below 18 years old, or are not residents of the Eastern Province, Saudi Arabia. These criteria were established to ensure that the participants in the study meet specific requirements and contribute to the accuracy and relevance of the research findings.

The questionnaire consists of personal information (general and demographic characteristics) such as sex, nationality, age, and other specific questions regarding the influence of selfie photographs and high expectations on the satisfaction rates among patients who had facial plastic surgery. The participants were requested to work individually in completing the questionnaire.

The data were analyzed using Statistical Packages for Social Sciences (SPSS) version 26 (Armonk, NY: IBM Corp., USA.). Categorical variables were elaborated as numbers and percentages. Continuous variables were shown as mean and standard deviation. The relationship between taking selfies and expectations toward cosmetic surgery among the socio-demographic characteristics of participants has been performed using the Chi-square test. Paired t-test was also performed to determine the differences in the satisfaction rate with appearance before and after cosmetic procedures. The cutoff point to determine statistical significance was set to less than p-0.05.

Research Ethics Committee, King Faisal University issued approval KFU-REC-2022-NOV-ETHICS282. Having reviewed the details submitted by the applicant regarding the above-named research project, the Research Ethics Committee at King Faisal University granted its ethical approval to the protocol.

## Results

This study enrolled 192 participants who underwent cosmetic procedures. As described in Table 1, 86 participants (44.8%) were aged between 18 to 30 years, with females being dominant 152 (79.2%). Most participants were Saudi nationals 181 (94.3%), and 86 (44.8%) were married.

**Table 1:** Socio-demographic characteristics of participants who underwent cosmetic procedures

Study data	N (%)^ (n=192)^
Age group	
18-30 years	86 (44.8%)
31-40 years	64 (33.3%)
41-50 years	28 (14.6%)
51-60 years	12 (06.3%)
>60 years	02 (01.0%)
Gender	
Male	40 (20.8%)
Female	152 (79.2%)
Nationality	
Saudi	181 (94.3%)
Non-Saudi	11 (05.7%)
Marital status	
Single	80 (41.7%)
Married	86 (44.8%)
Divorced	20 (10.4%)
Widowed	06 (03.1%)

**Figure 1:**
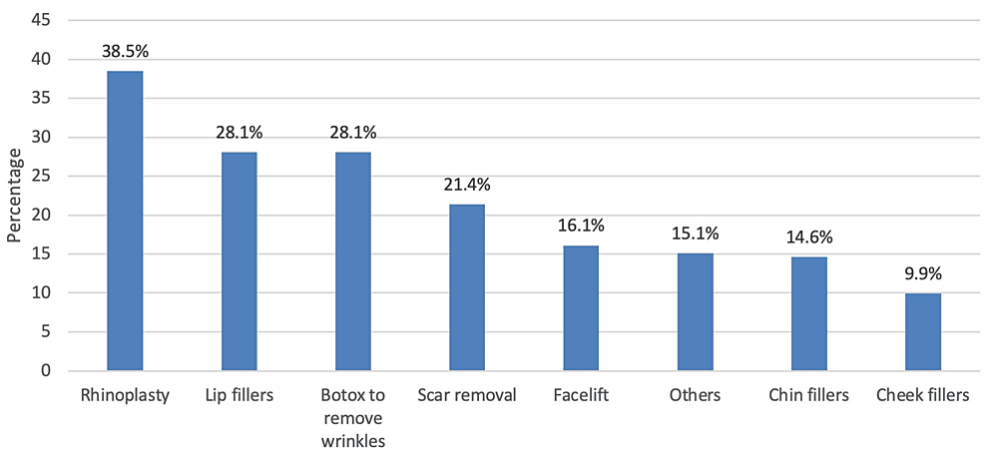
Type of cosmetic surgery being sought

In Figure 1, the most common type of cosmetic surgery being sought was rhinoplasty 74 (38.5%), followed by lip fillers 54 (28.1%) and botox to remove wrinkles 54 (28.1%).

**Figure 2:**
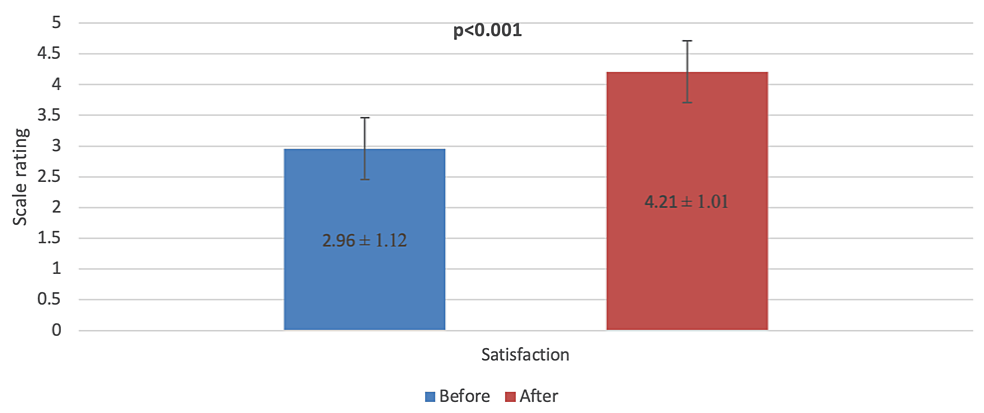
Comparison of satisfaction with selfies before and after cosmetic procedures

In Figure 2, it was observed that participants' satisfaction with selfies after cosmetic procedures was statistically significantly higher than before the intervention (p<0.001).

Table 2 shows that 57 (29.7%) underwent repeated procedures. Approximately 75 (39.1%) indicated that plastic surgery was better than the expectation. Nearly all subjects 181 (94.3%) were using social networks, and 137 (71.4%) were usually taking selfies. 

**Table 2:** Satisfaction with selfies after cosmetic procedures † Variable with multiple response answers.

Statement	N (%) ^(n=192)^
Have you ever repeated the same cosmetic procedure?	
Yes	57 (29.7%)
No	135 (70.3%)
Was plastic surgery what you expected?	
Better than my expectation	75 (39.1%)
Same as my expectation	80 (41.7%)
Less than my expectation	37 (19.3%)
Do you use social networks?	
Yes	181 (94.3%)
No	11 (05.7%)
Do you take selfies?	
Yes	137 (71.4%)
No	55 (28.6%)
Attitude toward taking a selfie ^(n=137)^	
Have your selfies increased after plastic surgery?	
Yes, I took more selfies after the operation	87 (63.5%)
No, say I take selfies after the operation	09 (06.6%)
There is no change before and after the operation	41 (29.9%)
How often do you take/take selfies after the plastic surgery?	
Once a month	25 (18.2%)
Once a week	28 (20.4%)
More than once a week	45 (32.8%)
Once a day	12 (08.8%)
More than once a day	27 (19.7%)
Do you use filters when taking a selfie?	
Yes	58 (42.3%)
No	25 (18.2%)
Sometimes	54 (39.4%)
When I take selfies ^†^	
I show deformities	26 (19.0%)
I face mocking from others	07 (05.1%)
I take many selfies	114 (83.2%)
Others	05 (03.6%)
Do you want/want another cosmetic procedure because of the selfies?	
Yes	48 (35.0%)
No	89 (65.0%)

When measuring the relationship between taking selfies with regard to the socio-demographic characteristics and expectations toward cosmetic procedures (Table 3), it was found that the prevalence of respondents who were taking selfies was significantly more common among the younger age group (p<0.001), unmarried (p=0.001) and those who were using social networking sites (p<0.001). However, taking selfies was significantly less prevalent among those who underwent facelift procedures (p=0.026). 

**Table 3:** Relationship between taking selfies among the socio-demographic characteristics and expectations toward cosmetic procedure † Some participants underwent more than one type of cosmetic procedure § P-value has been calculated using the Chi-square test. ‡ P-value has been calculated using independent sample t-test. ** Significant at p<0.05 level.

Factor	Taking selfie		P-value ^§^
	Yes N (%) ^(n=137)^	No N (%) ^(n=55)^	
Age group			
≤30 years	73 (53.3%)	13 (23.6%)	<0.001 **
>30 years	64 (46.7%)	42 (76.4%)	
Gender			
Male	30 (21.9%)	10 (8.2%)	0.567
Female	107 (78.1%)	45 (81.8%)	
Marital status			
Unmarried	86 (62.8%)	20 (36.4%)	0.001 **
Married	51 (37.2%)	35 (63.6%)	
Type of cosmetic procedure ^†^			
Rhinoplasty	58 (42.3%)	16 (29.1%)	0.088
Lip fillers	42 (30.7%)	12 (21.8%)	0.218
Cheek fillers	16 (11.7%)	03 (05.5%)	0.192
Chin fillers	23 (16.8%)	05 (09.1%)	0.172
Botox to remove wrinkles	36 (26.3%)	18 (32.7%)	0.369
Facelift	17 (12.4%)	14 (25.5%)	0.026 **
Scar removal	30 (21.9%)	11 (20.0%)	0.772
Have you ever repeated the same cosmetic procedure?			
Yes	42 (30.7%)	15 (27.3%)	0.643
No	95 (69.3%)	40 (72.7%)	
Was the plastic surgery what you expected?			
Better than my expectation	53 (38.7%)	22 (40.0%)	0.808
Same as my expectation	56 (40.9%)	24 (43.6%)	
Less than my expectation	28 (20.4%)	09 (16.4%)	
Do you use social networks?			
Yes	136 (99.3%)	45 (81.8%)	<0.001 **
No	01 (0.70%)	10 (18.2%)	
Satisfaction rate with the appearance before surgery (mean ± SD)	2.91 ± 1.12	2.76 ± 1.15	0.435^‡^
Satisfaction rate with the appearance after surgery (mean ± SD)	4.15 ± 1.04	4.22 ± 1.08	0.699^‡^

In Table 4, it was revealed that respondents who had high expectations about plastic surgery procedures were significantly more common among the older age group (p=0.008) and married participants (p=0.019). In addition, an increased satisfaction rate with appearance after surgery was more associated with high expectations of cosmetic surgery (p<0.001).

**Table 4:** Relationship between plastic surgery expectation among the socio-demographic characteristics and satisfaction with self-appearance * Respondents who had the same expectation were excluded from the analysis. † Some participants underwent more than one type of cosmetic procedure. § P-value has been calculated using the Chi-square test. ‡ P-value has been calculated using independent sample t-test. ** Significant at p<0.05 level.

Factor	Plastic surgery expectation * ^(n=192)^		P-value ^§^
	High N (%) ^(n=75)^	Low N (%) ^(n=37)^	
Age group			
≤30 years	25 (33.3%)	22 (59.5%)	0.008 **
>30 years	50 (66.7%)	15 (40.5%)	
Gender			
Male	16 (21.3%)	07 (18.9%)	0.766
Female	59 (78.7%)	30 (81.1%)	
Marital status			
Unmarried	33 (44.0%)	25 (67.6%)	0.019 **
Married	42 (56.0%)	12 (32.4%)	
Type of cosmetic procedure ^†^			
Rhinoplasty	26 (34.7%)	17 (45.9%)	0.248
Lip fillers	21 (28.0%)	10 (27.0%)	0.914
Cheek fillers	12 (16.0%)	03 (08.1%)	0.249
Chin fillers	15 (20.0%)	04 (10.8%)	0.223
Botox to remove wrinkles	23 (30.7%)	09 (24.3%)	0.485
Facelift	15 (20.0%)	06 (16.2%)	0.629
Scar removal	16 (21.3%)	06 (16.2%)	0.521
Have you ever repeated the same cosmetic procedure?			
Yes	24 (32.0%)	09 (24.3%)	0.402
No	51 (68.0%)	28 (75.7%)	
Satisfaction rate with the appearance before surgery (mean ± SD)	2.81 ± 1.17	2.57 ± 1.04	0.281 ^‡^
Satisfaction rate with the appearance after surgery (mean ± SD)	4.55 ± 0.90	3.05 ± 1.05	<0.001 ** ^‡^

## Discussion

This study is sought to determine the influence of high expectations and taking selfies on satisfaction with facial plastic surgery in the Eastern Region of Saudi Arabia. The outcome of this study showed that high expectation has a positive association with the satisfaction of self-appearance after cosmetic procedures, but taking selfies was observed to have no direct association with satisfaction. These findings are comparable with the study of Alkarzae et al. [14]. According to their reports, many of their subjects indicated satisfaction with their appearance after taking selfies, and 37.8% wished to undergo such procedures, specifically for people below 40 years old. However, in a study conducted among young British women [15], viewing images of people who had undergone cosmetic enhancement procedures seems to increase the desire of young women to undergo cosmetic surgery, particularly women who spent a significant amount of time using social media, subscribed to too many accounts and had low satisfaction with their appearance. Regardless of taking selfies, high expectations toward plastic surgery greatly influence self-appearance satisfaction post-cosmetic enhancement. In return, respondents' dissatisfaction with self-appearance could yield lower expectation rates with cosmetic procedures. 

Data in our study suggest that high expectations about plastic surgery were more prevalent among older age and married participants. However, there was no difference in expectations according to gender, the types of cosmetic procedures being sought, and undergoing repeated cosmetic procedures. In the UK [7], the greater likelihood of undergoing the cosmetic procedure was associated with low self-esteem, low self-rated attractiveness, low life satisfaction, few religious beliefs, and longer screen time on television. A study conducted in India [16] experimentally tested whether taking and posting selfies, with and without photograph retouching, increased the desire to undergo cosmetic surgery. The study noted an increase in social anxiety, a decrease in confidence, a decrease in physical attractiveness, and an increase in the desire to undergo cosmetic procedures seen in the experimental group, wherein these feelings were higher in women than men. However, in China [17], results suggest that selfie editing positively influences the consideration of cosmetic enhancement procedures. These associations were mediated by self-objectification and facial dissatisfaction. Implying that the use of photo editing applications seems to be a risk factor for women's self-appearance concerns resulting in consideration for cosmetic surgery. Hence, the author suggested limiting the use of photo editing applications to reduce the consideration of plastic surgery among women. 

Compared to satisfaction with a selfie before cosmetic surgery, the satisfaction rate with self-appearance was statistically significantly higher post-cosmetic intervention (p<0.001). In Iran [13], the decrease in desire for cosmetic rhinoplasty was associated with practicing more strict Islamic veiling techniques, which also increased women's body satisfaction, self-esteem, and psychological conditions. Interestingly, in Riyadh [18], the appearance of before and after photographs on social media platforms greatly affected women's decision to undergo rhinoplasty, with 52.7% of them disclosing that their decision to undergo cosmetic procedures was influenced by the advertisements seen on social media platforms. 

Nearly three-quarters of our subjects (71.4%) were taking selfies. Being younger, unmarried, and social media subscribers were more prevalent in taking selfies than the rest of the participants. Furthermore, the attitude of our respondents toward taking selfies increased after the facial enhancement procedure, as 63.5% of those who were selfies proponents indicated taking more selfies after the intervention with a frequency of more than once per week (32.8%). However, 42.3% expressed that they are still using filters when posting selfies, and as a result, more than one-third (35%) would want to repeat the cosmetic procedure. Several papers documented that taking selfies influences women to undergo cosmetic enhancement. For example, Dossari et al. [19], reported that 50.6% and 47.6% of their subjects believed that taking a selfie and using filters may have significant roles in deciding to undergo blepharoplasty. This has been concurred by the paper of Aldosari [20], with both taking selfies and filters increasing the desire to get cosmetic procedures, particularly among women, while in a study by Alkarzae et al. [14], dissatisfaction with self-appearance in selfies was the main reason of the sample population to undergo cosmetic enhancement procedures, wherein 5.8% had already undergone such procedures due to negative thoughts about their selfies. Taking selfies and filters may increase the demand for cosmetic procedures; however, it is necessary to raise awareness about the risks of the procedures. Therefore, authorities should exert more effort to educate the public about the risks posed by aesthetic surgeries. Simultaneously, it is important to lift spirits, particularly among individuals with low self-esteem, to prevent them from seeking cosmetic enhancement and reduce the number of cosmetic procedures subsequently undertaken.

Limitations of the study

There are certain limitations in the present research that should be acknowledged as important points to consider. This study has self-reported data that are not independently verified and it does not specify participants cities. The participants of this study were predominantly females (79.2%). In this research, we did not inquire about any changes in the usage of filters postoperatively.

## Conclusions

There was an increased satisfaction rate with self-appearance after plastic surgery. This results in high expectations for cosmetic procedures among end users. Furthermore, respondents who were married and more mature were more likely to exhibit better expectations of plastic surgery than other study populations. However, taking selfies may not directly influence expectations and satisfaction rates. This study provides evidence that high expectations with self-appearance yielded better satisfaction with plastic surgery, but taking selfies may not. This study's findings necessitate further investigations; therefore, a longitudinal study is warranted to establish the influence of high expectations and taking selfies after cosmetic enhancement procedures.
